# The change of bacterial community structure helped *Salvia miltiorrhiza* alleviate the pressure of drought stress

**DOI:** 10.3389/fpls.2025.1642597

**Published:** 2025-07-30

**Authors:** Hai Wang, Chen Wu, Xiaoyu Li, Hongmei Jia, Zhuyun Yan

**Affiliations:** ^1^ College of Medical Technology, Chengdu University of Traditional Chinese Medicine, Chengdu, China; ^2^ State Key Laboratory of Southwestern Chinese Medicine Resources, Chengdu University of Traditional Chinese Medicine, Chengdu, China; ^3^ School of Ethnic Medicine, Chengdu University of Traditional Chinese Medicine, Chengdu, China

**Keywords:** *S.miltiorrhiza*, drought stress, plant-microorganism interaction, PGPR, plant resistance

## Abstract

**Introduction:**

Drought stress poses a significant threat to plant growth and development, thereby adversely impacting agricultural productivity and ecosystem stability. In recent years, increasing attention has been given to plant–microorganism interactions as a promising strategy to enhance plant resistance to abiotic stresses.

**Methods:**

In this study, we evaluated the effects of microbial inoculation on the growth, photosynthetic performance, nutrient uptake, and root morphology of Salvia miltiorrhiza under drought stress. Microbial community composition was also analyzed to explore the interaction between drought stress and rhizosphere microbiota.

**Results:**

Our results demonstrated that microbial inoculation significantly alleviated the adverse effects of drought stress on S. miltiorrhiza. Inoculated plants exhibited a 3.61-fold increase in biomass compared to the uninoculated controls. Chlorophyll content increased by approximately 85.45%, while nitrogen and potassium contents rose by 27.77% and 33.27%, respectively. Furthermore, microbial inoculation improved root system architecture. Drought stress altered the rhizosphere microbial community, with the relative abundance of Enterobacteriaceae increasing by 5.50% and Brucellaceae decreasing by 2.76%.

**Discussion:**

These findings suggest that microorganisms can enhance plant drought resistance through multiple mechanisms, including the promotion of growth, nutrient absorption, and root development, as well as modulation of microbial community structure. This study provides a theoretical foundation and practical insights for the development of microbial-based strategies to improve plant resilience under drought conditions.

## 1 Introduction

Due to their immobility, plants are constantly exposed to a range of abiotic stresses, including drought, high temperatures, and salinity, all of which can significantly impair their growth and development, ultimately leading to reduced crop productivity (Zhang et al., 2021; Waadt et al., 2022; Nawaz et al., 2023). Among these, drought is considered one of the most severe constraints on plant performance and agricultural output worldwide (Farooq et al., 2009; Gupta et al., 2020). The accelerating pace of climate change has increased both the frequency and intensity of drought events, posing growing threats to plant health and yield (Yuan et al., 2023; Zeng et al., 2023). Substantial evidence indicates that drought stress adversely affects early plant growth by reducing parameters such as leaf size, leaf area, shoot length, and leaf dry weight (Zhang et al., 2018; He et al., 2024; Petek-Petrik et al., 2024). These growth limitations are closely linked to the disruption of vital physiological processes, particularly photosynthesis and carbon assimilation. Under drought conditions, plants tend to close their stomata to minimize water loss, which concurrently limits the uptake of atmospheric CO_2_. This results in reduced intercellular CO_2_ concentration, decreased stomatal conductance, and a subsequent decline in carbon fixation capacity (He et al., 2024; Singh, 2024; Wang and Dang, 2024). In response to drought, plants have evolved various adaptive mechanisms. Reduced water availability also limits the efficiency of nutrient uptake from the soil, further constraining plant growth (Meisser et al., 2019; Shinde et al., 2024). To adapt to water-deficient conditions, plants often allocate more resources to root development, resulting in an increased root-to-shoot ratio and deeper root systems, which enhance water and nutrient acquisition efficiency (Cheraghi et al., 2023; Khan et al., 2024; Shi et al., 2025). In summary, drought stress compromises leaf water content and water potential by inhibiting photosynthesis and nutrient uptake, ultimately suppressing leaf development and biomass accumulation. Therefore, improving plant drought tolerance has become a key focus in agricultural research.

In recent years, increasing attention has been directed toward plant–microbe interactions in the context of enhancing plant resilience to abiotic stress. In particular, microorganisms have been widely recognized for their critical role in improving plant drought tolerance (Trivedi et al., 2020; Pang et al., 2022; Huang et al., 2024). In the ever-changing environment, the recruitment of microorganisms by plants changes the composition and function of plant-related microbiomes, which ultimately provides an important guarantee for the survival and healthy growth of plants (Santoyo, 2022; Muhammad et al., 2025). Beneficial microbes contribute to plant adaptation to drought through multiple mechanisms, including the secretion of phytohormones, enhancement of nutrient uptake, and activation of antioxidant defense systems (Bhat et al., 2022; Wan et al., 2024). Endophytic bacteria can not only improve the length and density of plant roots to enhance plant drought tolerance, but also increase the content of osmotic regulators and activate antioxidant enzyme systems to indirectly help plants resist drought stress (Wang et al., 2022). Inoculation with arbuscular mycorrhizal fungi and plant growth-promoting bacteria has been shown to significantly enhance tobacco growth and biomass under drought conditions, primarily by increasing chlorophyll content, photosynthetic activity, and photosystem II efficiency (Zeng et al., 2025). In addition, plant growth-promoting bacteria promote the absorption of water and nutrients and promote the growth of branches by inducing the growth of plant roots and buds, thus alleviating the pressure of plant drought stress (Sharma et al., 2023; Kumar et al., 2024). Therefore, a deeper understanding of how changes in microbial community structure influence plant drought tolerance is essential for harnessing the functional potential of beneficial microbes to mitigate the adverse effects of drought stress.

*Salvia miltiorrhiza* is a traditional Chinese medicinal herb widely recognized for its pharmacological properties, including promoting blood circulation, removing blood stasis, unblocking meridians, relieving pain, cooling the blood, and reducing inflammation. However, in recent years, climate change has severely affected both the yield and quality of cultivated *S. miltiorrhiza*. Previous studies have demonstrated that drought stress significantly reduces chlorophyll content in leaves, aboveground biomass, root dry weight, and the accumulation of key bioactive compounds such as tanshinone IIA, leading to a supply that fails to meet clinical demand (Liu et al., 2011; Wei et al., 2017; Jiang et al., 2019). In this study, tissue-cultured seedlings of *S. miltiorrhiza* and their associated cultured bacterial communities were used as experimental materials. A co-culture system of plants and bacteria was established under drought stress conditions. High-throughput 16S rRNA sequencing was employed to characterize the composition and diversity of root-associated bacterial communities. By integrating data on plant growth parameters, photosynthetic performance, nutrient accumulation, and root morphology, we systematically assessed the role of bacterial communities in mitigating drought stress in *S. miltiorrhiza*. This research aims to clarify the relationship between root microbial community structure and the stress resilience and productivity of *S. miltiorrhiza*, thereby providing a theoretical basis for ecological cultivation strategies and offering valuable insights into the use of microbial technologies to enhance plant performance under stress conditions.

## 2 Materials and methods

### 2.1 Experimental design, sample collection and treatment

**Figure 1:**
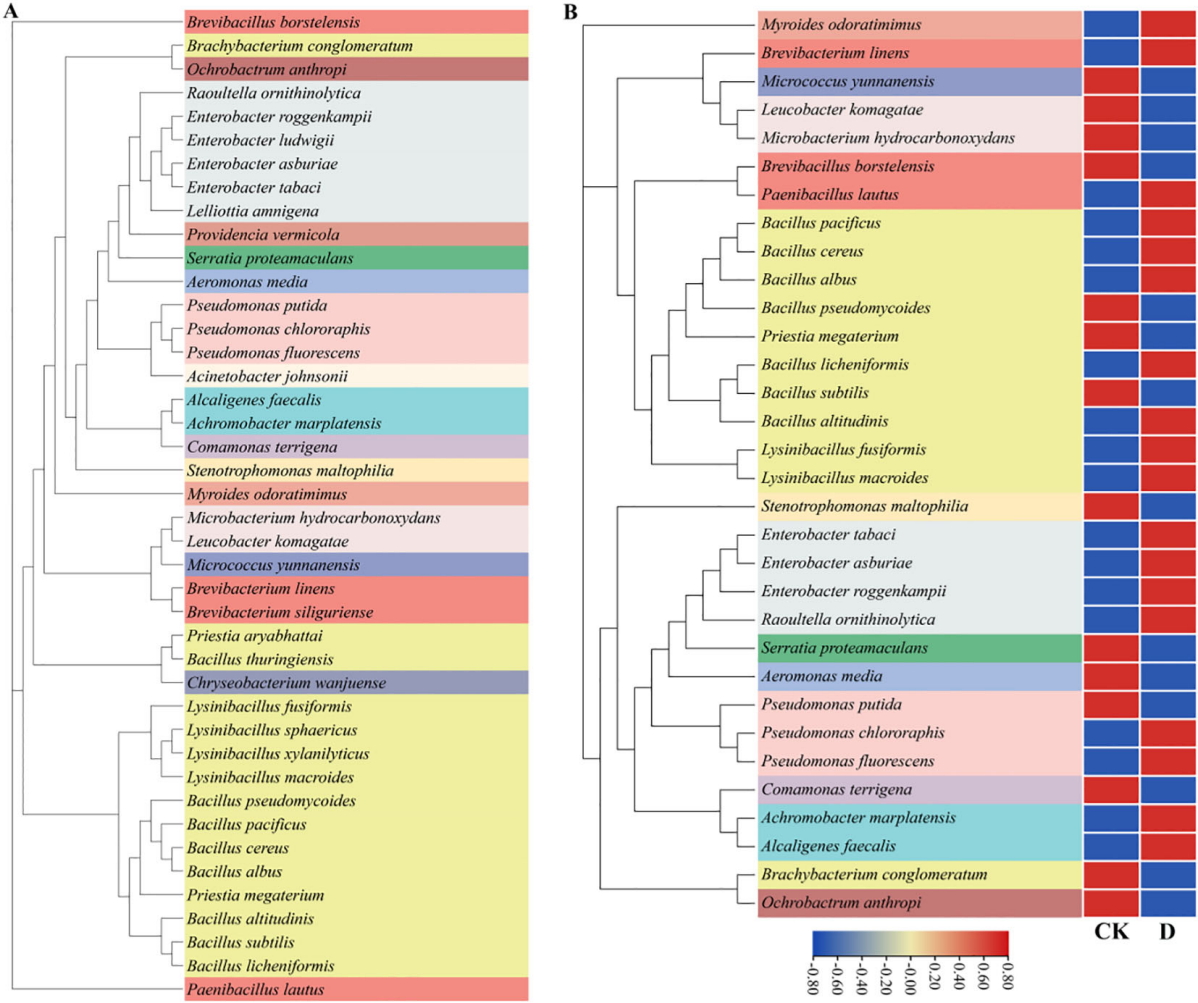
Phylogenetic trees of tested and treated bacterial strains annotated at the species level. CK was the blank group and D was the drought treatment. **(A)** The phylogenetic tree of all tested strains; **(B)** The heatmap of the relative abundance of bacterial species under drought stress.

Explants derived from newly developed leaves of large-leaf *S. miltiorrhiza* were used to initiate tissue culture seedlings (Jia et al., 2024). To closely mimic the native growth environment of *S. miltiorrhiza*, shale rocks collected from Ximeishan Village, Shiquan Township, Zhongjiang County, Sichuan Province were crushed to a particle size of 1–5 mm, dry-heat sterilized at 180 °C for 4 hours, and then used as the cultivation substrate. Bacterial cultures were prepared by growing individual strains in Luria-Bertani liquid medium at 28 °C with shaking at 180 rpm for 24 hours, and the cultures were subsequently adjusted to OD_600_ of 0.6–0.7 before use. The experimental bacterial inoculum was composed of a mixed suspension of all tested strains. A solid microbial inoculant was subsequently prepared by mixing this suspension with dry heat-sterilized shale powder, which had a particle size of less than 1 mm and was derived from crushed shale collected from Ximeishan Village, at a final concentration of 3 × 10^6^ CFU/g. The tested strains are presented in **Figure 1A**. Each seedling was applied with 20 g solid bacterial agent, and no bacterial agent was applied as the uninoculated group.

After 30 days of growth in pots, drought stress (D) was imposed by maintaining the substrate moisture content at 30%, while the control group (CK) was maintained at 70% substrate moisture. The growth temperature was set at 25 °C during the day and 20 °C at night, under a 16 h light/8 h dark photoperiod. The corresponding inoculated groups were designated as B-D (inoculated+drought) and B-CK (inoculated+control). Each treatment included six biological replicates. After 15 days of drought treatment, *S. miltiorrhiza* seedlings were harvested. After gently shaking off the substrate, seedlings were rinsed with sterile water and blotted dry using sterile filter paper. Fresh weight was recorded, and the plants were then separated into shoots and roots. The samples were immediately placed into sterile EP tubes, flash-frozen in liquid nitrogen, and stored at -80 °C for subsequent analyses.

### 2.2 Determination of physiological and biochemical indexes

#### 2.2.1 Determination of biomass

The total fresh weight (TW) of each *S. miltiorrhiza* seedling was measured by gently removing the plant from the growth substrate, rinsing the roots with sterile water to eliminate surface debris, blotting off excess water using sterile filter paper, and immediately weighing the whole plant (including both aboveground and underground parts) on an analytical balance.

#### 2.2.2 Determination of N/P/K content

The underground part was ground at 105°C for 15 min, and dried at 65°C to constant weight for mineral element analysis. H_2_SO_4_-H_2_O_2_ digestion, distillation method for the determination of total N content, molybdenum antimony anti colorimetric method for the determination of total P content, flame photometer method for the determination of total content (Duan et al., 2024).

#### 2.2.3 Determination of chlorophyll content

The chlorophyll content in leaves was determined by acetone colorimetry. Briefly, 0.2 g of fresh leaf tissue was homogenized in 10 mL of 80% (v/v) acetone and kept in the dark at 4 °C for 24 hours. The extract was centrifuged at 5,000 rpm for 10 minutes, and the absorbance of the supernatant was measured at 645 nm and 663 nm using a UV-visible spectrophotometer. Chlorophyll a, chlorophyll b, and total chlorophyll contents were calculated according to the method described by Croft (Croft et al., 2017).

#### 2.2.4 Determination of photosynthetic parameters

Photosynthetic parameters were measured using a LI-6400X portable photosynthesis system (LI-COR Biosciences, USA), following the manufacturer’s instructions. The net photosynthetic rate (Pn, μmolCO_2_·m^-2^·s^-1^), stomatal conductance (Gs, mmolH_2_O·m^-2^·s^-1^), intercellular CO_2_ concentration (Ci, μmolCO_2_·mol^-1^), and transpiration rate (Tr, mmolH_2_O·m^-2^·s^-2^) of the leaves were determined.

#### 2.2.5 Analysis of root morphology

*S. miltiorrhiza* seedlings were carefully removed from the pots, and any remaining substrate adhering to the roots was gently removed. The roots were then rinsed thoroughly with sterile water, blotted dry using sterile filter paper, and scanned using a root scanner (ScanMaker i800 Plus, Microtek, Taiwan) to capture high-resolution images of root morphology. The acquired root images were analyzed using the Wanshen LA-S series image analysis system to quantify root morphological parameters, including total root length, total root surface area, root volume, and the number of root tips.

### 2.3 DNA extraction, amplicon sequencing and data processing

According to the method established in our previous work (Jia et al., 2025), DNA was extracted from the root powders of *S. miltiorrhiza* using the E.Z.N.A^®^soil DNA Extraction Kit (OmegaBio-Tek, USA) as per the manufacturer’s instructions, and the NanoDrop2000 ultraviolet-visible spectrophotometer (ND2000, Thermo Scientific) was adopted. DNA purity and concentration quality tests were conducted in DE, USA. The V5-V7 region of the 16S rRNA gene was amplified using primers 799F (5’-AACMGGATTAGATACCCKG-3’) and 1193R (5 ‘-ACGTCATCCCCACCTTCC-3’), DNA integrity was evaluated via gel electrophoresis, with a representative image shown in **Supplementary Figure S1**. The presence of distinct and intact bands confirms that the extracted genomic DNA was of high quality and suitable for subsequent analyses. The purified PCR products were sequenced on the Illumina MiSeq PE300 high-throughput sequencing platform (Majorbio, China). The FASTP software (v0.20.0) was used for the quality control of the original sequencing sequence. The double-ended sequencing data were stitched according to overlap using the FLASH software (v1.2.7), and the chimerism was removed using the QIIME software (v1.9.1) to obtain the optimized sequence. The Blast Zone tool in TBtools software (v1.12) was used to compare the self-built library with the target sequence, and the species annotation table was obtained based on the highest matching value.

In order to evaluate the interactions among microorganisms, the bacterial OTUs obtained through annotation was used to construct the Spearman correlation network. The Spearman correlation coefficient r >0.6 is a positive correlation, r < -0.6 is a negative correlation, and p < 0.05 is a significant correlation. Among them, the OTU is set as a node, and the interaction relationship serves as an edge. The size of a node indicates the degree of node connection. Spearman’s correlation was calculated using the R package “Hmisc” and visualized by Gephi (v 0.10).

### 2.4 Data statistics and analysis

All data were analyzed by univariate analysis (one-way ANOVA) using SPSS 25.0 software, analyzed of variance and multiple comparisons using independent sample T-test and LSD method (p<0.05), plotted using Excel 2016 software and GraphPad prism 9.

## 3 Results

### 3.1 The root bacterial microbiome alleviated the stress of plants under drought stress

#### 3.1.1 Changes of biomass and photosynthetic parameters of *S. miltiorrhiza* under drought stress

**Figure 2:**
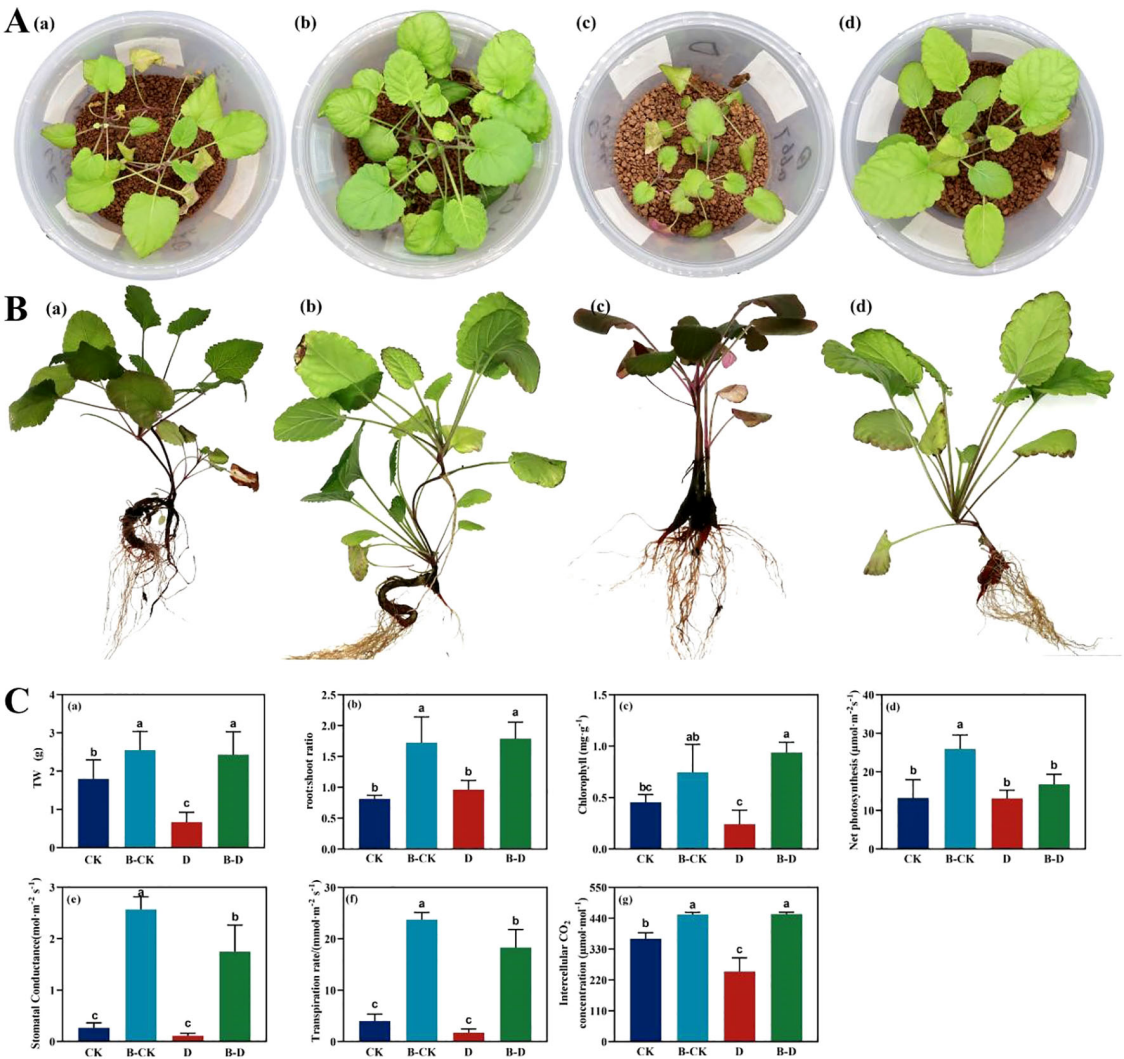
Changes of biomass and photosynthetic parameters of *S. miltiorrhiza* under drought stress. CK and B-CK were respectively the uninoculated group and the inoculated blank group, while D and B-D were respectively the uninoculated group and the inoculated drought stress group. **(A)** The growth status of *S. miltiorrhiza* under stress for 15 days; **(B)** The state of *S. miltiorrhiza* at the time of sample collection; **(C)** The changes of physiological and biochemical indicators of *S. miltiorrhiza* under drought stress: (a) TW is the total fresh weight; (b) root: shoot ratio; (c) The content of chlorophyll; (d) Net photosynthesis (μmolCO_2_·m^-2^·s ^-1^); (e) stomatal conductance (mmolH_2_O·m^-2^·s^-1^); (f) Transpiration rate (mmolH_2_O·m^-2^·s^-2^); (g) Intercellular CO_2_ concentration (μmolCO_2_·mol^-1^). Different lowercase letters indicate significant differences (p<0.05).

By observing the growth and development of *S. miltiorrhiza* seedlings under drought stress (**Figures 2A-C**), it can be known that the *S. miltiorrhiza* under drought stress is the smallest, with the fewest leaves, and the leaves turn yellow, indicating that it may be facing nutritional stress simultaneously. After inoculation with bacteria, the number of leaves increases, and the leaf area significantly increases (**Figures 2A-D**). Under uninoculated treatment, compared with CK, drought stress treatment significantly reduced the fresh weight of *S. miltiorrhiza*, which was approximately 37.4% of CK. Inoculation with bacteria significantly increased the biomass of the CK group and had a significant alleviating effect on D stress. It could significantly increase the total weight of *S. miltiorrhiza*, approximately 3.61 times that under stress. In the uninoculated treatment, compared with CK, drought stress treatment had no significant effect on its root-shoot ratio, chlorophyll content, Pn, Gs, and Tr. Inoculation with bacteria significantly increased the root-shoot ratio, chlorophyll content, Gs, Tr and Ci of *S. miltiorrhiza* under drought treatment, increasing by approximately 85.45%, 288.09%, 1463.06%, 956.00% and 81.82% respectively. Meanwhile, by comparing the indicators of CK under inoculation treatment with those under drought stress, it was found that under drought stress, the biomass of *S. miltiorrhiza* showed a downward trend, and chlorophyll and net photosynthetic rate significantly decreased. This suggests that utilizing microorganisms is an effective way to increase plant yield, and bacteria can alleviate the drought stress pressure encountered by plants.

#### 3.1.2 The changes of nitrogen, phosphorus and potassium contents in *S. miltiorrhiza* leaves under drought stress

**Figure 3:**
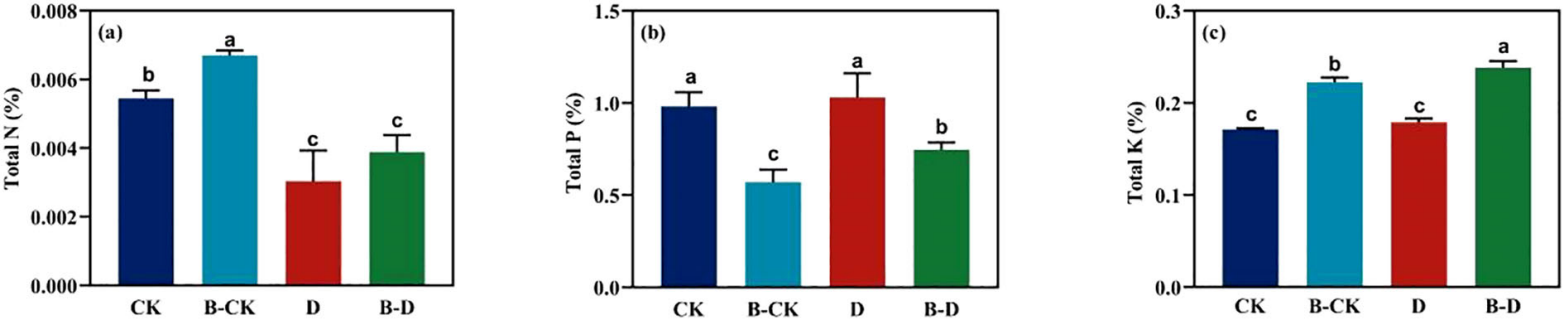
Changes of nitrogen, phosphorus and potassium in *S. miltiorrhiza* leaves under drought stress. CK and B-CK were respectively the uninoculated group and the inoculated blank group, while D and B-D were respectively the uninoculated group and the inoculated drought stress group. **(a)** N content in the leaves; **(b)** P content in the leaves; **(c)** K content in the leaves. Different lowercase letters indicate significant differences (p<0.05).

Under the uninoculated treatment, compared with the blank group, treatment D significantly reduced the N content in *S. miltiorrhiza* leaves by approximately 44.17%. Compared with the uninoculated group, the microbiome increased the contents of N (**Figure 3a**) and K (**Figure 3c**) in the leaves of *S. miltiorrhiza* under the same treatment by approximately 27.77% and 33.27% respectively, while significantly reducing the content of P (**Figure 3b**) in the leaves of *S. miltiorrhiza* under the same treatment. By comparing the indicators of CK under inoculation treatment with those under drought stress, it was found that under drought stress, the content of N in the leaves of *S. miltiorrhiza* significantly decreased, while the contents of P and K significantly increased. The above results suggest that microorganisms can promote the accumulation of two types of nutrients, N and K, but may promote the absorption and utilization of P element.

#### 3.1.3 The morphological changes of *S. miltiorrhiza* root system under drought stress

**Figure 4:**
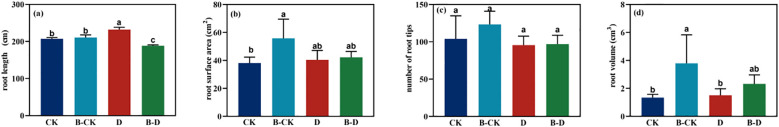
Changes in the root morphology of *S. miltiorrhiza* under drought stress. CK and B-CK were respectively the uninoculated group and the inoculated blank group, while D and B-D were respectively the uninoculated group and the inoculated drought stress group. **(a)** root length (cm); **(b)** root surface area (cm^2^); **(c)** number of root tips; **(d)** root volume (cm^3^). Different lowercase letters indicate significant differences (p<0.05).

In the B-CK group, the total root length (**Figure 4a**) and the number of root tips (**Figure 4c**) of *S. miltiorrhiza* were both higher than those in the CK group, and the root surface area (**Figure 4b**) and root volume (**Figure 4d**) were significantly increased, approximately 1.46 and 2.82 times that of the CK group. In the uninoculated group, the total root length, root surface area and root volume of *S. miltiorrhiza* showed an increasing trend under D stress compared with the CK group. In the inoculated group, the total root length, root surface area and root volume of *S. miltiorrhiza* under drought stress all showed a decreasing trend compared with the B-CK group. The above results indicate that under drought stress, plants adapt to the stress by promoting root development.

### 3.2 Drought stress drives the reassembly of the root bacterial microbiome

#### 3.2.1 The influence of drought stress on microbial composition

**Figure 5:**
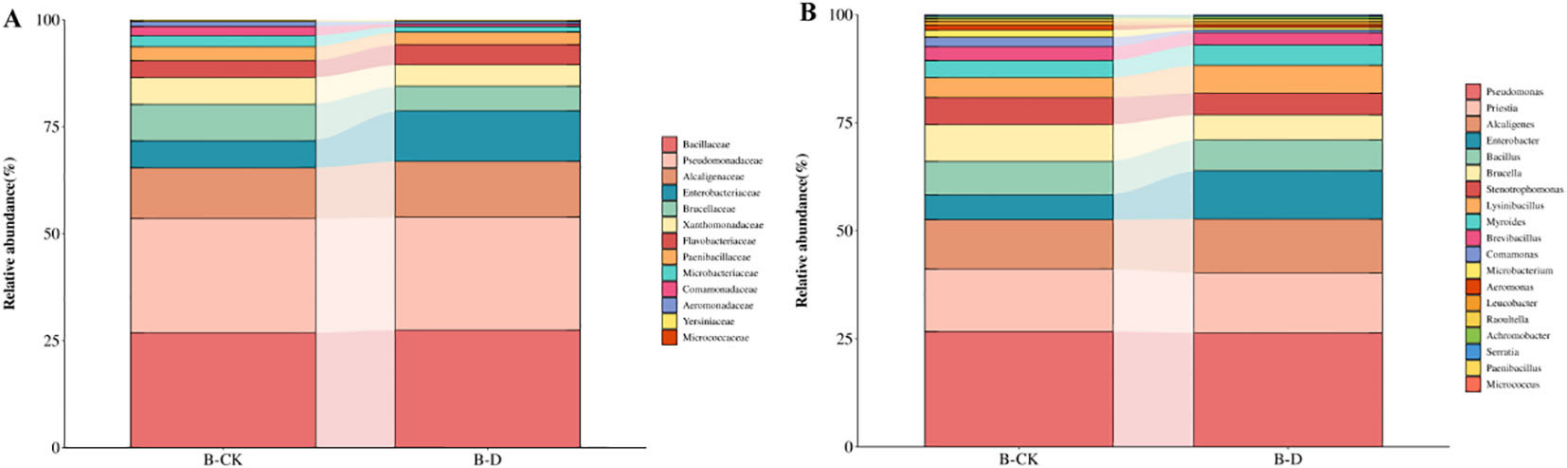
Changes at the family and genus levels of bacteria under drought stress. B-CK was the blank group and B-D was the drought treatment. **(A)** Changes in the relative abundance of bacteria at the family level; **(B)** Changes in the relative abundance of bacteria at the genus level.

A total of 42 species of bacteria belonging to 23 genera and 17 families were inoculated in this experiment. Through blast comparison with the local microbiome database, the compared bacteria were annotated to 33 species belonging to 19 genera and 13 families (**Figure 1B**). Comparing the differences at the family level between CK and group D, it was found that there was a total of 6 families with a relative abundance change of more than 1%. Compared with CK, the relative abundances of Enterobacteriaceae and Alcaligenaceae increased, while those of Comamonadaceae, Microbacteriaceae, Flavobacteriaceae and Xanthomonadaceae decreased. Among them, the relative abundance of Enterobacteriaceae increased most significantly, approximately by 5.50%, while the relative abundance of Brucellaceae decreased most obviously,approximately by 2.76% (**Figure 5A**). By comparing the differences at the genus level between group CK and group D, it was found that there was a total of 7 genera with a relative abundance change of more than 1%. Compared with group CK, the relative abundances of Alcaligenes, *Enterobacter* and *Lysinibacillus* increased. The relative abundances of *Comamonas*, *Microbacterium*, *Brucella* and *Stenotrophomonas* decreased. Among them, the relative abundance of *Enterobacter* increased most significantly, approximately by 5.42%, while the relative abundance of Brucella decreased most obviously, approximately by 2.76% (**Figure 5B**).

By comparing the differences at the species level between CK and group D, it was found that there was a total of 8 species with a relative abundance change of more than 1%. Compared with CK, the relative abundance of *Alcaligenes faecalis*, *Enterobacter asburiae*, *Enterobacter roggenkampii* and *Lysinibacillus macroides* increased. The relative abunences of *Comamonas terrigena*, *Microbacterium hydrocarbonoxydans*, *Ochrobactrum anthropi* and *Stenotrophomonas maltophilia* decreased. Among them, the relative abundance of *Enterobacter roggenkampii* increased most significantly, approximately increasing by 3.72%, while the relative abundance of *Ochrobactrum anthropi* decreased most obviously, approximately reducing by 2.76%. As can be seen from the above, under drought stress, the bacterial community undergoes adaptive recombination to help plants alleviate the stress.

#### 3.2.2 The influence of drought stress on microbial networks

**Figure 6:**
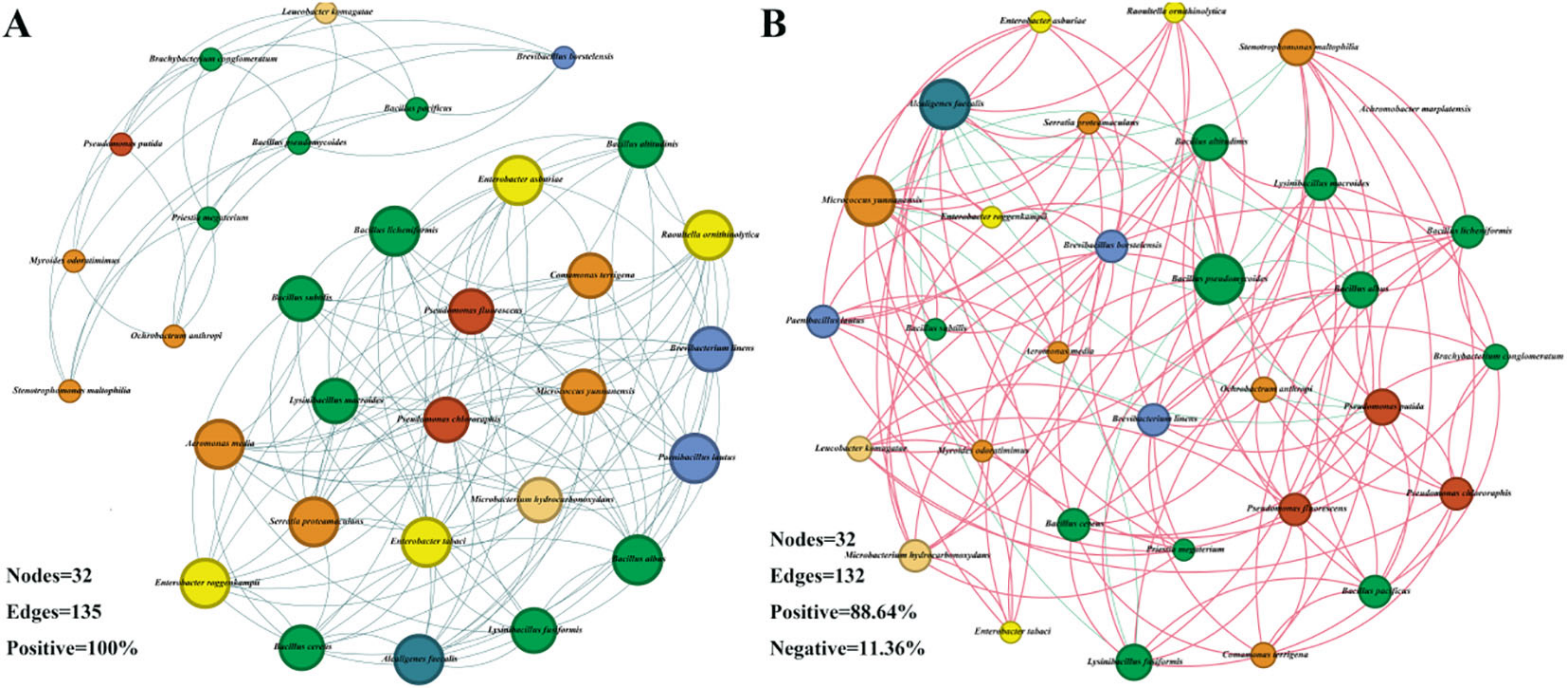
Microbial co-occurrence networks under different treatments. **(A)** Microbial co-occurrence network in the CK group; **(B)** Microbial co-occurrence network in Group **(D)** The size of each node is proportional to the degree and is colored at the family level of the microorganisms.

**Figure 7:**
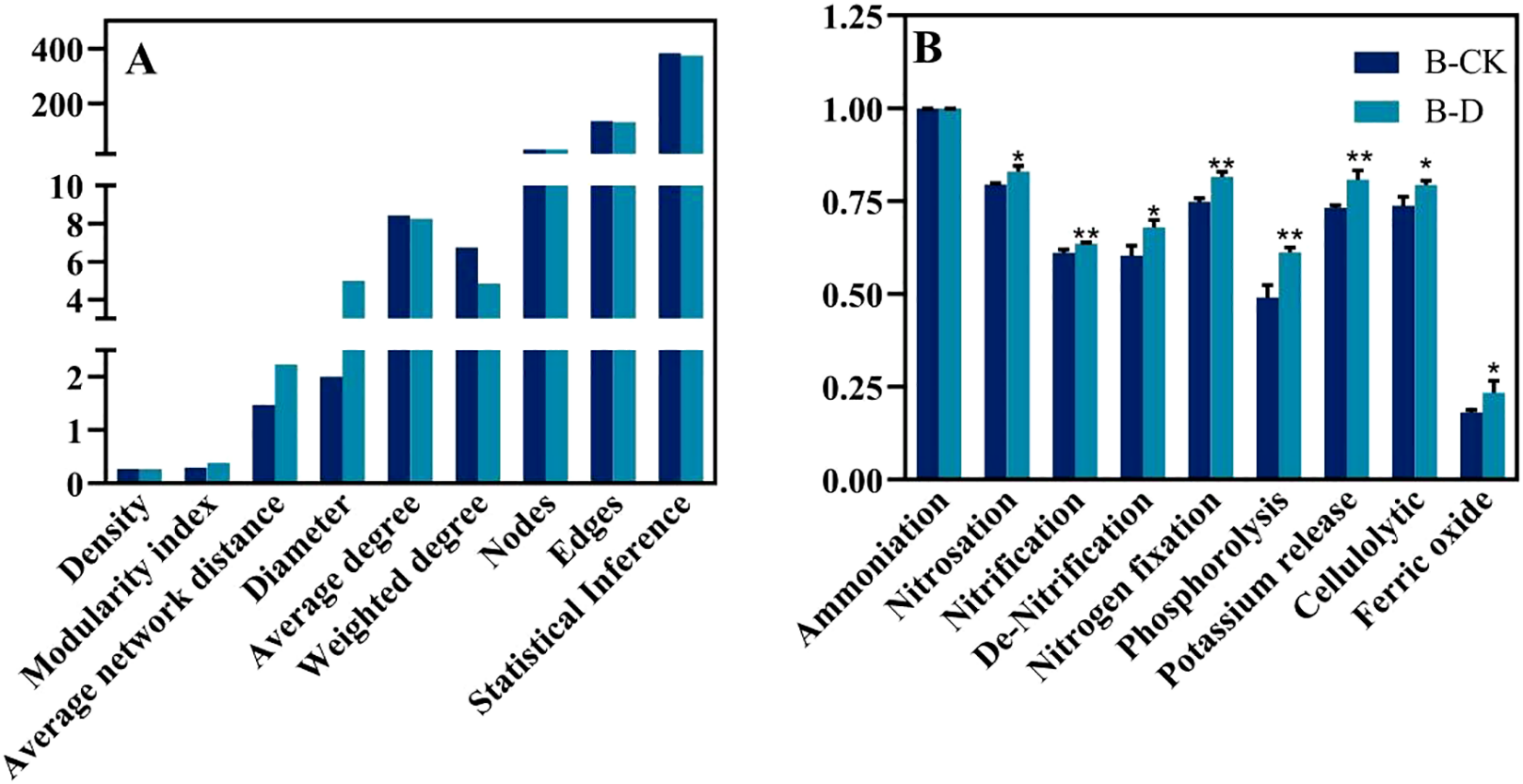
Changes of microbial co-occurrence network topology index and microbial function under different treatments. **(A)** Microbial co-occurrence network topology indexes; **(B)** Changes in microbial function.

To explore the effects of drought stress on the microbial community of *S. miltiorrhiza*, based on the Spearman correlation among OTUs, a microbial co-occurrence network was constructed with points and edges that were significantly (*p*<0.05) strongly correlated (| r | >0.6) to evaluate the potential microbial interactions under stress treatment. The red line and the green line represent the positively correlated side and the negatively correlated side respectively. In a co-occurrence network, positive correlation usually indicates a symbiotic relationship, while negative correlation indicates a competitive or predatory relationship. Compared with the control group, drought stress reduced the number of edges (**Figure 6**), decreased density, average degree, weighted degree and statistical Inference, and simultaneously reduced the proportion of positively correlated edges. However, the Modularity index, Average network distance and Diameter have been improved. It indicates that the competitive effect among microorganisms under drought treatment is stronger than that in the control group (**Figure 7A**). By comparing the composition of key nodes in the network, it was found that the connections between each node in the CK group were relatively tight, while under the D treatment, the main central nodes were *Alcaligenes faecalis*, *Bacillus pseudomycoides*, and *Micrococcus yunnanensis*. It indicates that bacteria can reorganize the interaction network among communities to help plants cope with stress.

#### 3.2.3 The influence of drought stress on microbial functions

Comparing the composition of functional microorganisms under CK and drought stress (**Figure 7B**), it was found that the relative abundance of bacteria with the functions of nitrosation, nitrification, de-Nitrification, nitrogen fixation, phosphorolysis, potassium release, cellulolytic and ferric oxide increased significantly compared with CK. It is suggested that plants under drought stress may enrich functional bacteria to enhance the utilization of nutrients to cope with stress.

## 4 Discussion

Drought stress is one of the most critical abiotic factors limiting plant growth and development, severely impairing physiological processes such as biomass accumulation, photosynthetic efficiency, and nutrient uptake. In recent years, plant–microbe interactions have emerged as a key regulatory mechanism enabling plants to withstand drought conditions. In this study, we systematically investigated the effects of drought stress on the growth, photosynthetic performance, nutrient accumulation, root morphology, and root-associated bacterial communities of *S. miltiorrhiza*. Our findings elucidate the role of beneficial microorganisms in mitigating drought stress and provide insights into the underlying mechanisms by which microbes enhance plant resilience under water-deficient conditions.

Under drought stress, the biomass of *S. miltiorrhiza* decreased significantly, the number of leaves decreased, and the leaves turned yellow, which was in line with the typical water stress symptoms reported in the literature, indicating that the drought stress modeling was successful in this experiment. Under drought conditions, the total fresh weight of the bacterial inoculation group was significantly increased, which was 3.61 times that of the uninoculated group, indicating that microorganisms can alleviate the negative effects of drought stress. In addition, drought stress significantly reduced chlorophyll content, Pn, Gs and Tr, indicating that drought stress suppressed photosynthetic activity. Nitrogen is the core component of chlorophyll, and potassium plays an important role in regulating stomatal opening and closing and water transpiration. Especially in arid environments, the role of potassium is more critical. It helps plants to maintain intracellular osmotic pressure and water balance, thereby improving drought resistance (Johnson et al., 2022; Querejeta et al., 2022; Hou et al., 2024). Under drought stress, the biomass and photosynthesis of *S.miltiorrhiza* were significantly improved after inoculation with microorganisms. At the same time, the phosphorus content in the leaves of *S.miltiorrhiza* was significantly reduced, while the nitrogen and potassium contents were significantly increased, indicating that bacteria preferentially help plants accumulate nitrogen and potassium elements that are more critical for water regulation and energy metabolism in leaves (Zhang et al., 2021). The relative reduction of phosphorus content, which is less supportive to photosynthesis, indicates that bacteria are more likely to distribute phosphorus to roots or other plant organs and reduce accumulation in leaves (Cao et al., 2024).

The change of root morphology is one of the key adaptation strategies for plants to cope with drought stress (Zahedi et al., 2025). In this study, drought stress significantly promoted the root development of *S.miltiorrhiza*, and root length, root surface area and root volume showed an increasing trend, confirming the view that the adjustment of root morphology enables plants to enhance water and nutrient acquisition through a larger root absorption area (Maurel and Nacry, 2020). The increase of root surface area and root volume in the microbial inoculation group indicated that microorganisms could alleviate the effects of drought stress on plants by improving the structure and function of roots and promoting the absorption of water and nutrients by plants (Bai et al., 2022).

The dynamic change of microbial community is the core of plant-microbe interaction (Xiong et al., 2021). In this study, 16S rRNA amplicon sequencing was used to analyze the effect of drought stress on the microbial community structure of root bacteria of *S.miltiorrhiza.* The results showed that drought stress significantly changed the composition of root microbial community. Enterobacteriaceae has been shown to promote the degradation of ACC, resulting in increased rice rhizosheath weight and plant growth under drought stress (Zhang et al., 2020). Alcaligenaceae under drought stress not only effect the population and activities of microorganisms inhabiting the rhizosphere but also various physiological and biochemical processes in plants that is. photosynthesis, respiration, translocation, uptake of ions, carbohydrates, and nutrient metabolism (Naseem et al., 2018). In this experiment, the relative abundance of Enterobacteriaceae and Alcaligenaceae increased significantly under drought stress, indicating that Enterobacteriaceae and Alcaligenaceae may be the core flora to resist drought stress.

Furthermore, we found that the interaction network between microbial communities changed significantly under drought stress. In the drought treatment group, the competition between microorganisms was enhanced, and the modularity of the network was improved, which indicated that microorganisms enhanced the adaptability of the community by adjusting the interaction mode under drought stress (Canarini et al., 2021; Xing et al., 2023). It is worth noting that the key nodes in the microbial community changed after drought stress, which were mainly dominated by some drought-resistant bacteria such as *Alcaligenes faecalis* and *Bacillus pseudomycoides*, indicating that these bacteria may play a central role in coping with drought stress. In addition, drought stress also promoted the increase of the abundance of functional dominant bacteria with nitrogen fixation, phosphorus solubilization and potassium solubilization, which indicated that the function of microbial community was optimized under drought stress to help plants acquire and utilize nutrients more effectively (Allsup et al., 2023; Ge and Wang, 2025).

## 5 Conclusions

In summary, this study revealed the complex mechanism of plant-microbe interaction under drought stress. Microorganisms significantly improved the drought tolerance of *S.miltiorrhiza* by adjusting the root microbial community structure, improving plant nutrient absorption, promoting root growth and optimizing the microbial interaction network. These results not only expand our understanding of plant-microbe interactions, but also provide theoretical support for the future development of microbial-based plant drought resistance strategies. Future research should further explore the synergistic effects of different microbial species and communities, and the differences in their roles in different plant species, so as to optimize the application of microorganisms to improve the drought resistance and productivity of agricultural crops.

## Data Availability

The original contributions presented in the study are included in the article/**Supplementary Material**. Further inquiries can be directed to the corresponding authors.

## Supplementary material

Supplementary Figure 1 Gel electrophoresis analysis of extracted DNA samples. Lanes 1–3 represent the control (CK) group, and lanes 4–6 represent the drought (D) treatment group. The PCR amplicon lengths ranged between 500 and 750 base pairs.
