# Supplementary figures and images for: The change of bacterial community structure helped Salvia miltiorrhiza alleviate the pressure of drought stress
Source: Front Plant Sci. 2025 Jul 30;16:1642597. doi: 10.3389/fpls.2025.1642597 (PMC12343674; doi:10.3389/fpls.2025.1642597)

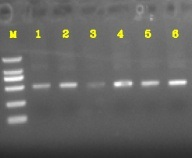

Supplement: Supplementary Figure 1 — Gel electrophoresis analysis of extracted DNA samples. Lanes 1–3 represent the control (CK) group, and lanes 4–6 represent the drought (D) treatment group. The PCR amplicon lengths ranged between 500 and 750 base pairs. [file Image1.png]
